# The Truth about Child Protection
**The Elements of Child Protection.* By Sigmund Engel. Translated from the German by Dr. Eden Paul. London: George Allen and Co., Ltd., 44 and 45 Rathbone Place. 1912. Price 15s. net.


**Published:** 1912-11-30

**Authors:** 


### THE TRUTH ABOUT CHILD PROTECTION.*

Mr. Sigmund Engel is a well-known jurist and official guardian of Budapest, and his work on Child Protection is familiar enough to experts. His translator, Dr. Paul, who has already done good work in bringing Dr. Bloch's writings before the English public, has given a full and accurate rendering of the book. It may be that many of us will differ radically on some points from the author. Dr. Engel takes the point of view of the Darwinist: he is a confirmed "Eugenist." In the excellent chapter dealing with the quality of the population, for example—a chapter which contains many home truths—he treats of punishment; he admits that the view that neither punishment nor reward should be employed as instruments of education is erroneous, but he refuses to accept corporal punishment. His arguments certainly deserve close attention.

He grounds his case on the following points: that such punishment is injurious to the child's health; that it is a sexual excitant; that it coarsens and hardens both teacher and pupil; that it tends to degrade the child; and, finally, that it makes the child vicious and deceitful. In a sense all these arguments apply to all punishments, and the case is not so much against corporal punishment as such, but against excessive and injudicious corporal punishment—a totally different thing. Once Dr. Engel's view is accepted that punishment is necessary, the nature and kind of punishment inflicted must be determined not on general but on particular and individual lines. Corporal punishment in one case may, as physicians are fully aware, lead to dire results; but such lamentable consequences are due largely to the indiscrimination of the teacher or parent, and must not be held to condemn the use of such punishment in general. The infliction of lines and tasks, and the curtailment of play, or, worse, the use of so-called moral punishments, may do incalculably more harm, physical and mental, to a sensitive child than a caning. In other chapters Dr. Engel is much more judicious. He points out the fallacy of taking it for granted that the children of inter-marriages are necessarily degenerates—a superstition which still lingers among the laity, and is even encouraged by some wild-talking Eugenists. There is no scientific reason why the child of parents who are close blood relations should not be normal provided always that the parents are normal and that the ancestry is untainted. We lack sufficient statistical data to prove this statement in its entirety, but there is ample evidence to support it and none to disprove it, though it is still an unpopular view. Dr. Engel is at some pains to deal fully with such interesting matters as marriage and heredity, prostitution, and crime in their relation to child protection. He insists that in cases in which one partner to a marriage suffers from disease divorce should be made as easy as possible, the interests of the children notwithstanding. From a purely scientific point of view this suggestion is quite fair, since it is precisely in the children's interest that such marriage should be dissolved. He shows that marriage prohibition as exercised to-day bears badly on some of the best potential stock, while it does not in the least interfere with the union of defective stocks who may procreate defective children. We can scarcely agree with him, however, that cripples and rachitic persons should be debarred from marriage, and we should want much more convincing evidence than we now possess to be justified in prohibiting the man who has suffered from gonorrhœa. Marriage prohibition has always led to a striking increase in the number of illegitimate births, and Dr. Engel gives an interesting proof of this. "In Bavaria, down to the year 1868, the local authorities imposed an unconditional veto upon the marriage of persons supported solely by wage earning: against this prohibition there was no appeal whatever. In the year 1868 most of the former marriage prohibitions were repealed. The sequel to this was that whereas from 1854 to 1868 illegitimate births constituted 22 per cent. of all births, in the seven years following 1868 the percentage of illegitimate births fell to 12.6 per cent."

To the school doctor, who has to be well acquainted with the problems of child protection, Dr. Engel's book is particularly interesting. Child mortality is dealt with fully. Statistical problems, artificial selection, education, fertility of the proletariat, pros and cons of child protection, the executive instruments, parental authority, the protection of illegitimate children, minors and guardianship, infant life protection, baby farming, women's labour and child labour, protection against disease, penal methods, punishable offences against children, and allied subjects are concisely and clearly discussed. A full and very informative chapter is devoted to the public elementary school and the particular problems it raises. A very interesting chapter is that devoted to child protection before, during, and immediately after birth, which is one that should be studied with care by every layman, and in which the author strongly urges the legal prohibition of the employment of pregnant women for at least two months before their confinement. In the same chapter the question of the insurance of motherhood is discussed. In the article dealing with offences against children the author deals with abortion, and inclines to the view that this should no longer be regarded as a punishable offence. The arguments advanced in support are very cogent; perhaps the most powerful is that, as Dr. Engel shows, the law is everywhere altogether inefficient, for it attacks not the act in itself but merely the poverty of the doer and the clumsiness of the execution. He admits that when the proposed reform is carried it will be necessary to make abortion, if effected by a married woman without her husband's consent, an adequate ground for divorce.

---

* *The Elements of Child Protection.* By Sigmund Engel. Translated from the German by Dr. Eden Paul. London: George Allen and Co., Ltd., 44 and 45 Rathbone Place. 1912. Price 15s. net.

We have said enough to indicate the scope and nature of the book, which deserves to be widely read and should get a permanent place on the practitioner's shelves. It is a pity that the published price of the work is so high that the sale of the translation must necessarily be limited, and we hope that the publishers will see their way clear to printing a cheap edition, which will be available for those who cannot afford high-priced text-books. Such a work, to do the most good, should be sold cheaply, for those who can afford the price charged at present can usually find the means to read the original and the authorities which are referred to in it.

